# *Correction: Helicobacter pylori *and gastroduodenal pathology: New threats of the old friend

**DOI:** 10.1186/1476-0711-7-6

**Published:** 2008-02-20

**Authors:** Niyaz Ahmed, Leonardo A Sechi

**Affiliations:** 1 Pathogen Evolution Group, Centre for DNA Fingerprinting and Diagnostics (CDFD), Hyderabad, India; 2 Department of Biomedical Sciences, University of Sassari, Sassari, Italy

## Abstract

Since publication of our article (Ahmed and Sechi: *Ann Clin Microbiol Antimicrob *2005, 4:1), we have noticed several errors.

## Correction

We lately discovered that due to an inadvertent and accidental mistake, our review/update [1] needs some important corrections; we profusely regret and offer our sincerest apologies and correct the text as under.

Please be aware that throughout this article the citations in round brackets refer to the reference list from the original manuscript [1], and the citations in square brackets refer to newly introduced citations that appear in the reference list at the end of this manuscript.

The following sentence should have been included at the end of the original abstract.

"This review is written with a fair interest to reproduce, through open access channel, a follow up and update of our previous detailed discussions on the subject (Carroll et al: *Infect Genet Evol*. 2004, 4:81–90)."

In '**The conundrum of strain diversity...**' section, the first three sentences should have read:

"Within the bacterial populations, genome content keeps recasting due to selection pressures, as a result genomic diversity increases; this facilitates adaptation of particular strains to permissive hosts. H. pylori with its highly diverse gene pool is therefore, a model for studying genome plasticity in the context of colonization of individual hosts (34)."

In the "**Link with cancer: the oncogenic cagA protein**" section, the eighth sentence onwards should read as follows.

"The phosphorylated CagA (51, 52) binds to a SHP2 phosphatase which acts as an oncoprotein (64). The phosphorylated CagA mediated derangement of SHP2 signaling is an important putative mechanism of *H. pylori *induced gastric carcinogenesis (64, 65). Based on type and abundance of tyrosine phosphorylation motifs (TPM) (52), CagA is classified as East Asian and Western types (64)'. East Asian type of CagA is regarded as a risk factor possibly due to its high biological activity (63, 64) and therefore, high incidence of gastric cancer in East Asian countries might be the reflection of this. One puzzling attribute of *H. pylori *is the differential outcome of infection and severity of inflammation in different populations of the world (63). Possible explanation for this might originate from the analysis of genetic (polymorphism of human genes encoding receptors for *H. pylori *adhesins), environmental (diet) and social factors (transmission dynamics). Among these, diversity of cagA in *H. pylori *strains might be involved in determination of the type and severity of disease. The East-Asian and Western forms of CagA possess structurally distinct tyrosine phosphorylation/SHP2-binding sites – EPIYA-D and EPIYA-C, respectively (64). Interestingly, the severity of inflammation, activity of gastritis, and the extent of atrophy were significantly higher in patients with gastritis who were infected with the East-Asian type cagA-positive strains as compared to patients who were infected either with cagA-negative or Western type cagA-positive strains (65). Gastric cancer mortality in Japan could be associated with large prevalence of East Asian type CagA containing isolates (65), thus explaining that patients colonized with such isolates might be at the highest risk of developing gastric cancer as compared to those who are colonized with Western type CagA containing *H. pylori*.

Among Western-CagA harboring strains, the numbers of EPIYA-C sites directly correlate with levels of tyrosine phosphorylation, SHP2-binding and morphological types of epithelial cells (hummingbird effect) (64). It has been described that the number of TPM of EPIYA-C (western) type determine the severity of atrophic gastritis and its outcome (cancer) in the cases of infection caused by strains that harbor Western type CagA (66)."

In the section "**The number-2 virulence determinant: vacuolating cytotoxin (*Vac*A) of *H. pylori***" sentences five-eight should have cited references (20, 2, 22).

"VacA protein, a secreted 95 kD peptide, varies in the signal sequence (alleles s1a, s1b, s1c, s2) and/or its middle region (alleles m1, m2) between different *H. pylori *strains (20, 21, 22). The different combinations of s and m regions determine the production of cytotoxic activity. Strains with the genotype s1 m1 produce high levels of vacuolating cytotoxin in vitro (20, 21, 22). Strains with the genotype s2 failed to produce detectable toxin (20)."

Sentence 13 should have cited new references.

"Among other functions, VacA is suggested to inhibit invariant chain (Ii)-dependent pathway of antigen presentation mediated by the MHC class II, and, to possibly induce apoptosis of epithelial cells [2,3]."

Sentence 14 to the end, should have read as follows.

"Apart from its well-known and widely discussed cytotoxic role (20, 21, 22), VacA has been shown to cause apoptosis [3] and inhibition of lymphocyte proliferation and thereby promotes a possible condition of local immunosuppression [4]."

In section **"Plasticity region cluster" **sentence five should not have referenced Figure 1.

"This type IV cluster is comprised of 7 genes, homologous to the *vir *B operon of *A. tumifaciens *carried in a 16.3 kb genomic fragment called tfs3."

In section "**Do we need to eradicate *H. pylori *from this earth?**" within the second paragraph, the second and third sentences should have cited new references [5,6] and read:

"In addition, the incidence of *H. pylori*-negative, non-NSAID peptic ulcer disease (PUD) (idiopathic PUD) is reported to increase with time. Also, it appears that *H. pylori*-positive ulcers are not necessarily the *H. pylori*-induced ulcers because there are two possible outcomes of the *H. pylori *induced pathology, first the existence of *H. pylori*-positive non-recurring ulcer and secondly, recurring ulcer after cure of *H. pylori *infection [5,6]."
